# Gender Differences in Health-Related Quality of Life in Patients with Systolic Heart Failure: Results of the VIDA Multicenter Study

**DOI:** 10.3390/jcm9092825

**Published:** 2020-08-31

**Authors:** Alberto Garay, Javier Tapia, Manuel Anguita, Francesc Formiga, Luis Almenar, María G. Crespo-Leiro, Luis Manzano, Javier Muñiz, José Chaves, Trinidad De Frutos, Pedro Moliner, Xavier Corbella, Cristina Enjuanes-Grau, Josep Comín-Colet, on Behalf of VIDA-IC Multicenter Study Investigators

**Affiliations:** 1 Unidad de Insuficiencia Cardiaca Comunitaria (UMICO), Servicio de Cardiología, Hospital Universitario de Bellvitge, L’ Hospitalet de Llobregat, 08907 Barcelona, Spain; alberto.garay.melero@gmail.com (A.G.); pmoliner@bellvitgehospital.cat (P.M.); cristinaenjuanes@gmail.com (C.E.-G.); 2 Programa Territorial de Atención Integrada a la Insuficiencia Cardiaca Comunitaria de la Gerencia Metropolitana Sur del Instituto Catalán de la Salud, Hospital Universitario de Bellvitge, L’ Hospitalet de Llobregat, 08907 Barcelona, Spain; 3 Unidad de Cardio-Oncología Hospital de Bellvitge–Instituto Catalán de Oncología, L’Hospitalet del Llobregat, 08907 Barcelona, Spain; 4 Grupo de Investigación en Enfermedades Cardiovasculares, Instituto de Investigación Biomédica de Bellvitge (IDIBELL), L’ Hospitalet de Llobregat, 08907 Barcelona, Spain; jtapia@bellvitgehospital.cat; 5 Departamento de Ciencias Clínicas, Universidad de Barcelona, 08907 Barcelona, Spain; 6 Unidad de Insuficiencia Cardíaca, Servicio de Cardiología, Hospital Universitario Reina Sofía, Córdoba 14004, Argentina; manuelanguita@secardiologia.es; 7 Servicio de Medicina Interna, Hospital Universitario de Bellvitge, l’ Hospitalet de Llobregat, 08907 Barcelona, Spain; fformiga@bellvitgehospital.cat (F.F.); xcorbella@bellvitgehospital.cat (X.C.); 8 Unidad de Insuficiencia Cardíaca y Trasplante, Servicio de Cardiología, Hospital Universitario La Fe, 46009 Valencia, Spain; lualmenar@gmail.com; 9 Unidad de Insuficiencia Cardiaca Avanzada y Trasplante Cardiaco, Servicio de Cardiología, Instituto de Investigación Biomédica de A Coruña (INIBIC), Complexo Hospitalario Universitario de A Coruña (CHUAC), SERGAS, Universidade da Coruña (UDC), 15006 A Coruña, Spain; Marisa.Crespo.Leiro@sergas.es; 10 Servicio de Medicina Interna, Hospital Universitario Ramón y Cajal, Departamento de Medicina y Especialidades Médicas, Facultad de Medicina y Ciencias de la Salud, Universidad de Alcalá, IRYCIS, 28034 Madrid, Spain; luis.manzano@uah.es; 11 Instituto Universitario de Ciencias de la Salud, Instituto de Investigación Biomédica de A Coruña (INIBIC), Universidade da Coruña, 15006 La Coruña, Spain; javmu@udc.es; 12 Centro de Investigación Biomédica en Red de Enfermedades Cardiovasculares (CIBERCV), 15705 Santiago de Compostela, Spain; 13 Medical Department, Internal Medicine, Pfizer Biopharmaceuticals Group, 28108 Alcobendas, Spain; jose.chaves@pfizer.com (J.C.); trinidad.defrutos@pfizer.com (T.D.F.); 14 Cátedra HESTIA en Atención Integrada Social y Sanitaria, Facultad de Medicina y Ciencias de la Salud, Universitat Internacional de Catalunya, Sant Cugat del Vallès, 08017 Barcelona, Spain; 15 Servicio de Cardiologia, Hospital Universitario de Bellvitge, L’ Hospitalet de Llobregat, 08907 Barcelona, Spain

**Keywords:** heart failure, gender, health related quality of life, generic and specific questionnaires of quality of life, real world evidence

## Abstract

Previous studies have shown that heart failure is associated with worse health-related quality of life (HRQoL). The existence of differences according to gender remains controversial. We studied 1028 consecutive outpatients with heart failure and reduced ejection fraction (HFrEF) from a multicentre cross-sectional descriptive study across Spain that assessed HRQoL using two questionnaires (KCCQ, Kansas City Cardiomyopathy Questionnaire; and EQ-5D, EuroQoL 5 dimensions). The primary objective of the study was to describe differences in HRQoL between men and women in global scores and domains of health status of patients and explore gender differences and its interactions with heart failure related factors. In adjusted analysis women had lower scores in KCCQ overall summary scores when compared to men denoting worse HRQoL (54.7 ± 1.3 vs. 62.7 ± 0.8, *p* < 0.0001), and specifically got lower score in domains of symptom frequency, symptoms burden, physical limitation, quality of life and social limitation. No differences were found in domains of symptom stability and self-efficacy. Women also had lower scores on all items of EQ-5D (EQ-5D index 0.58 ± 0.01 vs. 0.67 ± 0.01, *p* < 0.0001). Finally, we analyzed interaction between gender and different clinical determinants regarding the presence of limitations in the 5Q-5D and overall summary score of KCCQ. Interestingly, there was no statistical significance for interaction for any variable. In conclusion, women with HFrEF have worse HRQoL compared to men. These differences do not appear to be mediated by clinical or biological factors classically associated with HRQoL nor with heart failure severity.

## 1. Introduction

Patients with chronic heart failure (HF) have a significant impairment in health-related quality of life (HRQoL), which is comparable or even worse than other chronic life-limiting diseases such as chronic renal failure on haemodialysis, previous stroke, or Alzheimer’s disease [1]. HF is associated with several symptoms and physical limitations that impacts significantly in HRQoL [1,2]. However, these effects on the perceived health status cannot be justified only by physical or biological factors, but other psychosocial and demographic factors are likely to have significant influence [3,4].

The VIDA-IC study was a Spanish multicenter study that assessed HRQoL in more than 1000 patients with systolic HF [1]. This study showed that most of the factors associated with poorer quality of life in HF were also clinical factors related to severity of HF, such as comorbidity burden, recent hospitalization, age, or New York Heart Association (NYHA) functional class. However, demographic factors, such as gender, also independently determine perceived health status. In this study, female gender was associated with poorer HRQoL independently of several clinical factors related to HRQoL [1]. However, other studies have yielded heterogeneous results, making the gender breach in HRQoL a controversial issue in the field of HF that needs further exploration [5,6,7,8,9,10,11,12,13].

The primary aim of this pre-specified analysis of the VIDA-IC Study was to confirm and describe in detail the existing gender differences in HRQoL between men and women analyzing the HRQoL data obtained from validated instruments to measure self-reported health status across global scores and specific domains. As a secondary objective, we aimed to explore and determine potential interactions on global HRQoL and its domains between HF-related factors and gender.

## 2. Methods

### 2.1. Study Design

The methodology of the VIDA-IC study has been previously reported [1,14]. Briefly, the VIDA-IC study was a national, cross-sectional, descriptive observational study conducted by 115 physicians throughout Spain (cardiology and internal medicine specialists) between October 2011 and January 2012. HRQoL was studied in consecutive patients with HF by using 2 questionnaires and the determining factors related to it were analysed. The study protocol was approved by the Ethics and Clinical Research Committee of the Hospital del Mar Medical Research Institute (IMIM) de Barcelona. Written informed consent was signed by all patients prior to inclusion in the study.

### 2.2. Study Population and Inclusion-Exclusion Criteria

Consecutive HF patients who were attended to the specialized outpatient clinic (Cardiology or Internal Medicine) and who met the following criteria were included: age ≥ 18 years, diagnosis of chronic HF with left ventricular ejection fraction (LVEF) ≤ 40% in the last 12 months and stable clinical condition. Exclusion criteria were: Patients waiting for heart transplant or valve surgery, inability to assess and complete HRQoL questionnaires, extra-cardiac disease with life expectancy less than 1 year, hospital admission of non-cardiovascular causes in the month prior to inclusion and hospitalization at the time of inclusion. Patient inclusion was stratified by the presence or absence of recent HF admission (<1 month and >6 months without HF admission) into a 1:1 ratio for each of the recruiting investigators. The information corresponding to the baseline data was obtained after informed consent, from the patient anamnesis or their medical history. For the present study, it was available complete information for 1028 patients out of 1037 of the VIDA-IC study participants.

### 2.3. Assessment of Patient-Centred Health Outcomes in Quality of Life

All study patients self-administered the Kansas City Cardiomyopathy Questionnaire (KCCQ) [15] and the EuroQol 5D (EQ 5D) [16]. The KCCQ is a specific tool for HF, composed by 23 items clustered in 7 dimensions. The score for each dimension has a theoretical range from 0 to 100 points, in which higher score reflects better health status. In addition, three summary scores are calculated. The symptom summary score as a result of addition of symptom frequency and severity (excluding stability). The clinical summary score as a result of addition of physical and symptom limitation domains. The overall summary score as a result of clinical summary and quality of life and social limitation domains. The EQ-5D is a generic instrument that consists of a visual analogue scale (VAS) with general health self-assessment and 5 domains (mobility, self-care, usual activities, pain/discomfort, and anxiety/depression). For VAS, the range is from 0 (worst state) to 100 (best state). Regarding the rest of dimensions, results can be expressed as overall summary index (EQ-5D index) or also can be expressed as a percentage of patients who report some type of impairment in each dimension. Both scales have been validated in Spain [16,17].

### 2.4. Statistical Analysis

Continuous variables were expressed as mean ± standard deviation. Categorical variables were expressed as *n* (percentage) and compared using the χ^2^ test. Continuous variables were compared using the independent t-test or the Mann-Whitney’s U-test when normal distribution could not be assumed. Univariate logistic regression models and univariate linear regression models including gender as an independent variable were conducted to assess the clinical and demographic factors associated with HRQL. Based on the univariate linear regression analyses, several multivariate models were performed with backward elimination method to determine which factors maintained an independent association with patient centred health outcomes, including gender as an independent variable. Using general linear models, gender-adjusted marginal means of different KCCQ and EQ-5D summary scores were obtained. The association of clinical factors with HRQoL level was explored in a gender-stratified mode using binary logistic regression models. Finally, gender interaction with those clinical variables with prognostic influence on the results in HRQoL questionnaire scores was explored using logistic regression and general linear models.

Multivariate models were adjusted for variables that showed association with HRQL or that have well-known prognostic influence on heart failure and reduced ejection fraction (HFrEF). These variables were gender, age, number of comorbidities, systolic blood pressure, heart rate, body mass index, NYHA functional class, LVEF, aetiology of HF, chronic kidney disease, hypertension, diabetes mellitus, anaemia, department where patient was recruited into the study, recent HF admission prior to inclusion, time since diagnosis, and disease-modifying HF medical treatment. A *p*-value < 0.05 was considered statistically significant. The statistical analyses were performed using SPSSv23 (version 23.0; IBM, Armonk, NY, USA) and Stata v11 (version 11.1; StataCorp LLC, TX, USA).

## 3. Results

The VIDA-IC study recruited 1037 patients. Only 9 patients (0.9%) were excluded from the present analysis due to incomplete data. The final cohort of this analysis comprised 1028 patients, most of them male (719, 70%) with a mean age of 71 ± 11 years. Table 1 shows the demographic and clinical characteristics of patients included in the study, both, overall and according to gender. Women were older than men, had a lower prevalence of ischemic heart disease, and lower overall burden of co-morbidities. In contrast, co-morbidities such as anaemia and obesity were more common in women than in men.

Overall, women compared to men reported worse quality of life represented by lower scores in the generic (EQ-5D) and disease-specific instruments (KCCQ). Table 2 shows the distribution of summary scores, dimensions, and domains of the KCCQ and EQ-5D quality of life questionnaires in the overall population and according to gender. Women reported limitations more frequently in all domains of the EQ-5D questionnaire and had lower scores in VAS compared to men. Similar results were obtained in the analysis of the KCCQ: women showed lower scores indicating more affected HRQoL in the domains informing on physical limitation, symptom burden, symptom frequency, quality of life, and social limitation.

**Figure 1:**
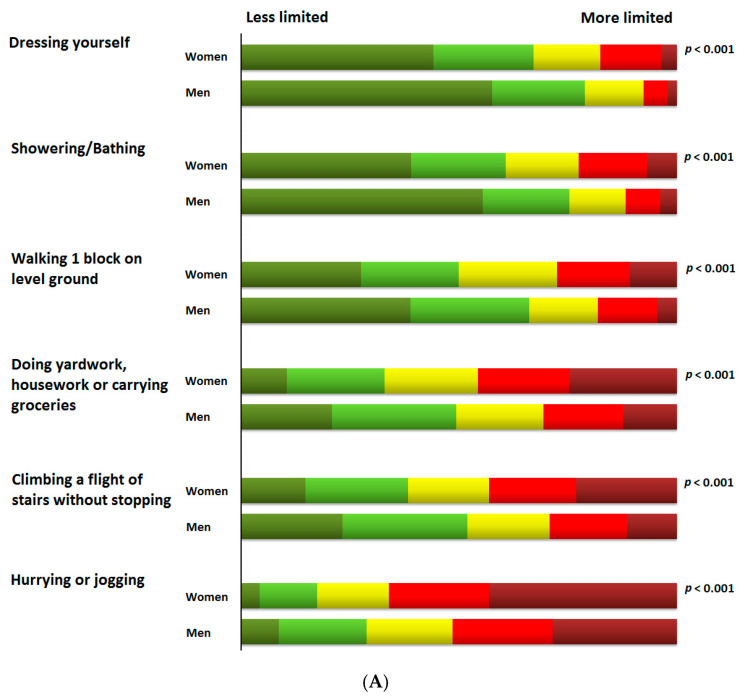

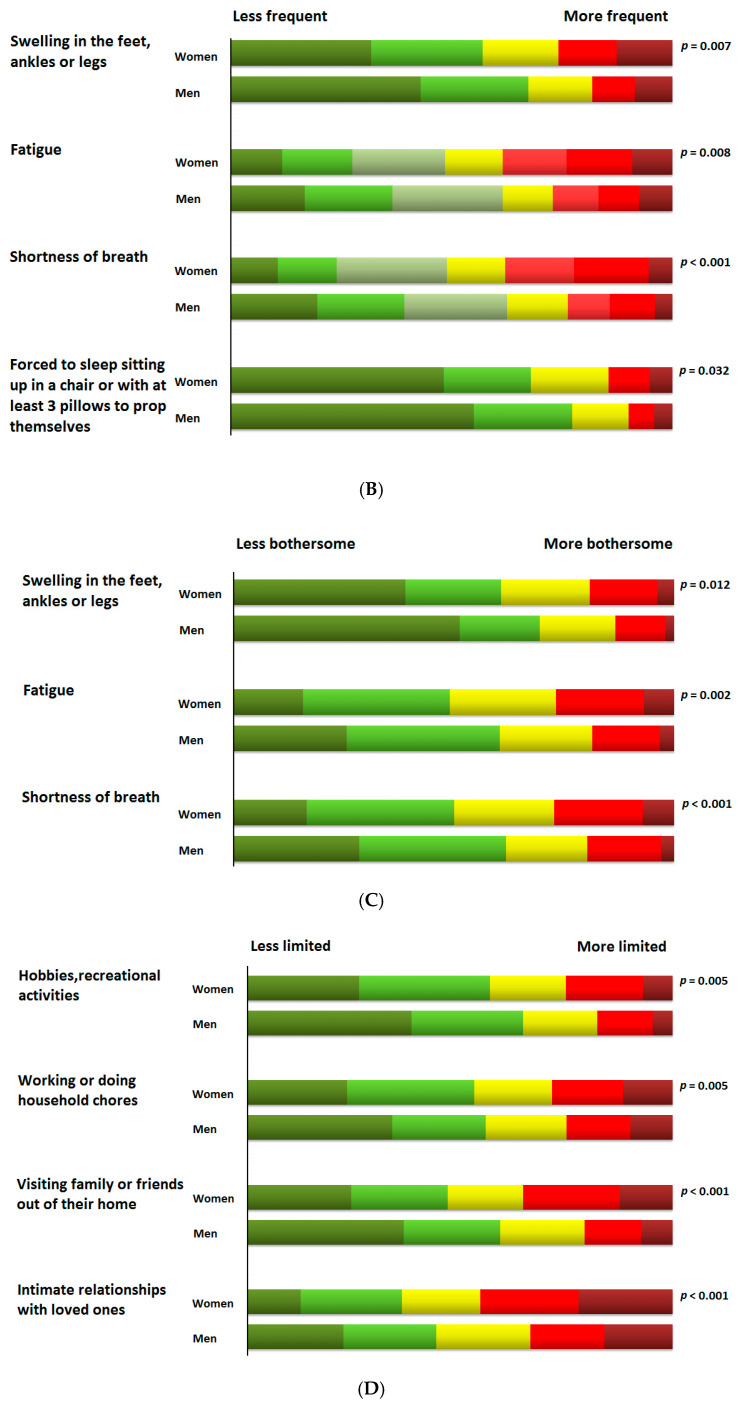
Percentage distribution of scores of the different KCCQ items in selected domains according to gender. (**A**) Physical limitation; (**B**) Symptom frequency; (**C**) Symptom burden; (**D**) Social limitation.

**Figure 2:**
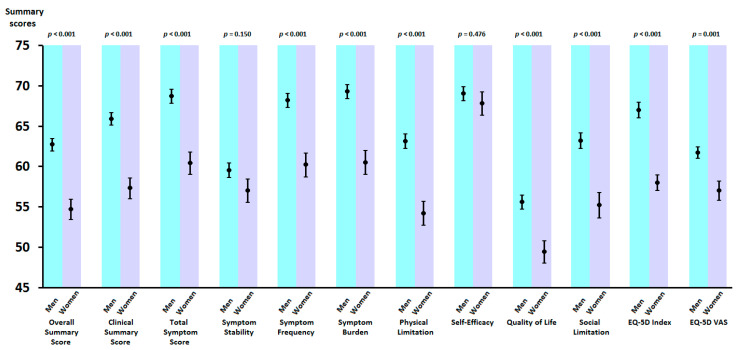
Adjusted scores (marginal means ± standard error of the mean) of the summary scores and sub-domains of the KCCQ and EQ-5D according to gender using multivariate general linear models. Adjustment variables were: age, body mass index, heart rate, NYHA functional class, LVEF, co-morbidity burden, HF etiology, presence of hypertension, diabetes mellitus, chronic kidney disease, atrial fibrillation, hemoglobin, admission service, recent admission for HF (last 6 months), and time since HF diagnosis.

Quality of life scores and their distribution for each of the items of the KCCQ in the whole cohort and according to gender are presented in Tables S1 and S2. Figure 1 shows the percentage distribution of scores of the different KCCQ items in selected domains according to gender. The burden imposed by HF experienced by women was more prominent than the reported by men in each of the items analysed across the domains informing on symptoms frequency, symptom burden, social limitations, and physical limitations with particular impact on usual activities such as getting dressed, showering or bathing, walking, doing housework or climbing stairs. In this regard, women (Table S2 and Figure 2) were more likely to report moderate to severe physical limitations (defined as scoring 1–3 at each individual item of the physical limitation domain of the KCCQ) compared to men in usual activities such as dressing up (43% more likely in women than in men, *p*-value < 0.001), showering, or bathing (45% more likely in women than in men, *p*-value < 0.001), walking (39% more likely in women than in men, *p*-value < 0.001), and doing housework (28% more likely in women than in men, *p*-value < 0.001). A similar trend was observed for individual items of the social limitation domain such as carrying out recreational activities (20% more likely in women than in men, *p*-value = 0.005), visiting family and friends (26% more likely in women than in men, *p*-value < 0.001), doing household chores (26% more likely in women than in men, *p*-value = 0.005) or having intimate relationships (16% more likely in women than in men, *p*-value < 0.001).

We used linear regression models to evaluate the role of gender as an independent variable on summary scores of generic and HF-specific instruments along with other demographic and clinical factors associated with HRQoL (Table S3). In adjusted models, female gender remained significantly associated with worse the overall HRQoL with any of the instruments evaluated (KCCQ OSS: standardized β coefficient = −0.144; *p*-value < 0.001; EQ-5D Index: standardized β coefficient = −0.157; *p*-value < 0.0001; EQ-5D VAS; standardized β coefficient = −0.106; *p*-value = 0.0005) and this association was independent of other significant determinants such as older age, advanced NYHA functional class, LVEF, the burden of co-morbidities, and recent HF admission.

Figure 2 shows adjusted scores (marginal means ± standard error of the mean) of the summary scores and sub-domains of the KCCQ and EQ-5D according to gender using general linear models. Women scored significantly lower on EQ-5D summary scores and all KCCQ sub-domains except for symptom stability and self-efficacy. Adjusted models included those variables that showed association with the KCCQ summary score (KCCQ OSS) in univariate linear regression models. In sensitivity analysis, the addition of systolic blood pressure and/or optimal medical treatment in HF did not change the results.

To further explore the interplay between gender and other clinical and biological factors in terms of self-perceived health status and particularly its effects on the burden imposed in important dimension and domains of HRQoL captured by the EQ-5D, we conducted binary logistic regression evaluating the association of such factors with the probability of reporting limitations in mobility, usual activities, self-care, pain/discomfort, or anxiety/depression. In unadjusted analyses, women reported more frequently limitations in mobility (OR 2.0, 95% CI (1.5–2.6); *p*-value < 0.0001), self-care (OR 2.0, 95% CI (1.5–2.6); *p*-value < 0.0001), usual activities (OR 1.8, 95% CI (1.4–2.4); *p*-value < 0.0001), pain/discomfort (OR 2.0, 95% CI (1.5–2.7); *p*-value < 0.0001) and anxiety/depression (OR 1.3, 95% CI (1.0–1.8); *p*-value < 0.029).

We conducted stratified analysis by gender of the clinical determinants of limitations in all the dimensions of EQ-5D (Table S4). In women (Table S4A), and in men (Table S4B), advanced NYHA class and higher burden of co-morbidities were significant determinants of reporting any limitation in all the domains of the EQ-5D. The chronic kidney disease, diabetes mellitus, anaemia, older age (except in anxiety/depression), and recent hospital admission were also associated with limitations in all dimensions of the EQ-5D in both genders although these associations only met the threshold for statistical significance in the men stratum.

**Figure 3:**
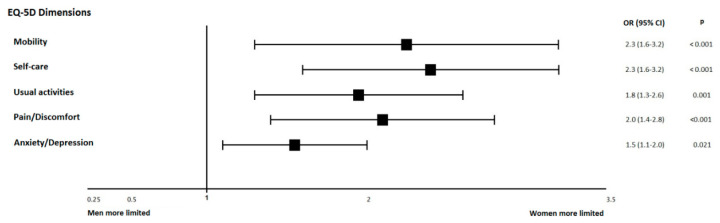
Multivariate binary logistic regression evaluating the association of gender with the probability of reporting limitations in the 5 dimensions captured in the EQ-5D (mobility, usual activities, self-care, pain/discomfort, or anxiety/depression). Results are representing odds ratios and 95% confidence intervals comparing women with men (reference category) of reporting any limitation in each of the dimensions of the EQ-5D questionnaire. Variables included in multivariate model were those factors that showed significant association with health-related quality of life (HRQoL) in univariate analyses.

Multivariate logistic regression analyses (Table S5 and Figure 3) confirmed that women, compared to men, were more likely to report limitations in mobility (OR 2.3, 95% CI (1.6–3.2); *p*-value < 0.0001), self-care (OR 2.3, 95% CI (1.6–3.2); *p*-value < 0.0001), usual activities (OR 1.8, 95% CI (1.3–2.6); *p*-value < 0.001), pain/discomfort (OR 2.0, 95% CI (1.4–2.8); *p*-value < 0.0001) and anxiety/depression (OR 1.5, 95% CI (1.1–2.0); *p*-value < 0.021). The models were adjusted for clinical covariates that showed significant association in univariate analyses. Interestingly, the negative impact of female gender was independent of the presence or absence of other important prognostic factors such as age, NYHA class, LVEF, chronic kidney disease, diabetes mellitus, anaemia, recent hospitalization and co-morbidity burden, among others.

Finally, in order to explain the differences found in HRQoL according to gender, we wanted to assess whether gender may be associated with significant interactions with other clinical determinants of HRQoL.

We first explored this approach to explain the limitations more frequently reported by women than in men in each domain of the EQ-5D. We, accordingly, conducted adjusted logistic regression analyses that included the interaction term between gender and each of the determinants explored in previous models. As shown in Table S6, no significant interactions were identified between gender and the remaining determinants explored.

Following these analyses, we aimed to assess whether KCCQ overall summary score gradients may be affected by potential interactions between gender and specific clinical factors associated with both HRQoL and prognosis. We used adjusted general linear multivariate models in order to analyse the effect of gender on the KCCQ OSS stratified according to key prognostic variables in patients with HFrEF such as LVEF, age, NYHA functional class, co-morbidity burden, HF aetiology, and recent hospitalization. Adjusted marginal means (±SEM) across genders and across key clinical factors along with *p*-values for the interaction terms explored are shown in Table 3. Consistently, at each key prognostic variable stratum, women always showed significantly lower scores on the KCCQ overall summary score (*p*-value beside each row). None of the interactions explored between gender and the above-mentioned key variables were significant (*p*-value in the bottom row).

## 4. Discussion

The main finding of this study is that in the setting of systolic heart failure, HRQoL is significantly and substantially worse in women compared to men across all domains of self-perceived health status. There are few studies that have analyzed the impact of gender on HRQoL in HF and most of them analysed small and heterogeneous samples [5,6,7,8,9,10,11,12,13]. Furthermore, these studies show conflicting results. So, it is important to highlight the main results of our study, identifying female gender that a clear and independent determinant of poorer HRQoL in this setting. The present study provides results from an extensive multicenter cohort of patients, so it provides important evidence to clarify this issue.

In addition, our study provides a detailed description about the existing gender differences in HRQoL between men and women. The impairment of self-reported health status in women involves all dimensions of the HRQoL including more pronounced limitations in physical, social, and symptoms domains compared to men. Furthermore, this difference in HRQoL according to gender was not determined by clinical and biological factors classically associated with HRQoL in HF: no differences were observed in the determinants of HRQoL across gender strata and no significant interactions were observed between gender and key clinical factors in terms of patient-reported outcomes. Moreover, differences in self-efficacy (self-care) or stability of HF cannot explain the differences observed between women and men in terms of HRQoL since the scores in the domains evaluating self-care and symptom stability did not differ according to gender. Overall, these findings suggest that other determinants may the actual drivers that could explain the HRQoL breach between women and men.

Previous research has shown that there are differences in the onset and impact of cardiovascular disease and HF according to gender [18,19,20,21]. Male gender is an independent cardiovascular risk factor and determines that cardiovascular disease develops at a younger age. On the other hand, the female gender is associated with a higher incidence of atypical symptoms and delays in diagnostic of cardiovascular conditions [22,23]. Overall, life expectancy is higher in women, but age-related comorbidities are also more prevalent [24]. Consequently, women tend to develop HF in older age, have more frequent HF with preserved LVEF, and ischemic aetiology is less prevalent compared to men [25,26,27]. Although women with HF have a longer life expectancy, there is evidence that suggests that their additional years of life are associated with more psychological and physical disability [8]. Therefore, the heart failure in women occurs in a different background than men. So, the women could have a different experience for the same disease.

Our results are in line with studies of HRQoL in other areas of cardiovascular disease such as atrial fibrillation or ischemic heart disease, in which it has been suggested that the female gender is also associated with worse HRQoL [28,29,30]. However, it has not been clarified whether these differences are a real consequence of gender-related factors or whether they are related to a different presentation of the disease according to gender [30].

Our work analyzes HF outpatients with reduced LVEF and does not only show that women have worse HRQoL, but also delves on involved factors. The differences observed in HRQoL according to gender can be mediated by differences in somatic factors (clinical or biological) or mediated by individual psycho-social factors [1,4,5,13]. Psychosocial factors, such as health literacy, self-care behaviours, years of education, cognitive function, functional social support, family role, dependency on basic and instrumental activities of daily living, caregiver status, affective status, and socioeconomic factors, among others, may be differentially expressed between women and men and, consequently, may potentially explain the gender gap on self-perceived health status.

We did not find any significant interaction between gender and somatic factors (clinical or biological) that help us to explain the gender differences found in patient-reported outcomes. These findings suggest that the clinical and biological HF-related factors classically associated with HRQoL are not sufficient to explain the HRQoL gap between men and women. Hence, other elements, such as psychosocial determinants, may well play a role in this setting. In this regard, we may hypothesize that the psychosocial consequences of physical limitation in HF, particularly in the elderly, could lead to a more pronounced loss of the social role in women compared to men and this, in turn, may help to explain the gender breach in terms of self-perceived health status. Interestingly, HF is an age-associated disease and, consequently, limitation in domestic and social activities could have an important weight, at least in certain cultures, in order to explain these differences. Beyond the above-mentioned, the additional hypotheses helping to interpret our findings focus on construct characteristics of the instruments used to evaluate patient-reported outcomes. We are not certain whether the structure and item wording of the instruments used for HRQoL assessment in our study may allow better capture of limitations in health status in women compared to men. Future studies are necessary to shed light on this matter and explore the role of psychosocial and socioeconomic factors as drivers of the gender gap in patient-reported outcomes among patients with HF.

### Limitations of the Study

This study has the intrinsic limitations of a cross-sectional evaluation. It does not provide information on longitudinal changes of health status or its dynamic interrelations with the clinical variables explored over time. It does not either allow drawing conclusions on true causality but merely explores the associations described. The population included in this study is representative of the subgroup of patients with HFrEF who are routinely attended in hospital outpatient settings. Hence, it is not possible to ascertain whether our findings could be extrapolated to different populations of patients with HF such as patients with HF and preserved LVEF or community-dwelling HF patients. Even though the population of the original VIDA study was included between October/2011 and January/2012, and in this time there have been therapeutic advances in the field of HFrEF, the clinical profile of patients with HFrEF has not shown important changes over time. The baseline characteristics of those patients included in our study are similar to other recent studies in HFrEF [31]. We think that the data from VIDA is still valid and applicable to the clinical practice for the purpose of this analysis.

Lastly, a comprehensive psychosocial and socioeconomic evaluation was not planned and performed in patients recruited in the VIDA-IC study. We do not know whether having included information on health literacy, social support, cognitive function, among others, would have attenuated the gender gap in self-perceived health status observed in our study. We hypothesize that these factors may play an important role and can help to explain the impaired HRQoL observed in women compared to men. Future research studies orientated to verify this hypothesis are needed.

## 5. Conclusions

In this multicenter study conducted with a large sample of patients with systolic HF, we have shown that women report worse HRQoL compared to men. The breach in self-perceived health status according to gender was consistent across all the domains and dimensions that define HRQoL and particularly those describing symptom burden, symptom frequency, physical and social limitations. We did not observe any significant interaction between gender and clinical or biological factors related to HF that could explain the above-mentioned differences observed between women and men. These findings may support the hypothesis that other aspects beyond somatic factors associated with HF, such as psychosocial or socioeconomic variables may be the actual drivers of the gender gap in patient-reported outcomes observed in our study. Future investigations are necessary to clarify the factors involved in the HRQoL in women with HF.

## Figures and Tables

**Table 1:** Demographic and clinical characteristics of patients included in the study, both overall and according to gender.

	*n*	Overall *n* = 1028	Women *n* = 309 (30%)	Men *n* = 719 (70%)	*p* Value
Age (years)	1015	71 ± 11	73 ± 10	70 ± 11	<0.0001
BMI (kg/m^2^)	997	27.7 ± 3,9	28.2 ± 4,4	27.5 ± 3.6	0.010
Systolic Blood Pressure (mmHg)	1022	127 ± 19	129 ± 20	126 ± 18	0.092
Heart rate (bpm)	1021	74 ± 16	76 ± 16	73 ± 15	0.017
NYHA Functional class I-II vs. III-IV	994	546 (54.9)/448 (45.1)	158 (53.4)/138 (54.6)	388(55.6)/310 (44.4)	0.531
Previous HF admission,	1028	860 (83.7)	262 (84.8)	598 (83.2)	0.581
HF admission <6 months,	1028	507 (49.3)	148 (47.9)	359 (49.9)	0.586
Time from diagnosis (years)	936	2.7 (0.9–5.9)	2.8 (0.9–6.3)	2.7 (0.9–5.7)	0.690
LVEF (%)	993	33.7 ± 6.8	34.4 ± 6.7	33.4 ± 6.8	0.027
Ischemic etiology of HF	1028	521 (50.7)	100 (32.4)	421 (58.6)	<0.0001
Comorbidities					
Hypertension	1028	815 (79.3)	240 (77.7)	575 (80.0)	0.403
Diabetes mellitus	1028	450 (43.8)	141 (45.6)	309 (43.0)	0.451
Previous AMI	1028	447 (43.5)	91 (29.4)	356 (49.5)	<0.0001
Dislipidemia	1028	704 (68.5)	196 (63.4)	508 (70.7)	0.023
Chronic kidney disease	1028	242 (23.5)	63 (20.4)	179 (24.9)	0.128
Atrial Fibrillation	1028	443 (45.5)	150 (51.5)	293 (43.0)	0.017
Anemia	1028	194 (18.9)	80 (25.9)	114 (15.9)	0.0003
COPD	1028	199 (19.4)	28 (9.1)	171 (23.8)	<0.0001
TIA/Stroke	1028	142 (13.8)	47 (15.2)	95 (13.2)	0.430
Anemia	1028	204 (19.8)	44 (14.2)	160 (22.3)	0.003
Cancer	1028	81 (7.9)	30 (9.7)	51 (7.1)	0.165
Chronic Hepatopathy	1028	43 (4.2)	7 (2.3)	36 (5.0)	0.060
Obesity	1028	239 (23.2)	98 (31.7)	141 (19.6)	<0.0001
Low weight	1028	13 (1.3)	6 (1.9)	7 (1.0)	0.227
Overweight	1028	801 (77.9)	239 (77.3)	562 (78.2)	0.806
Sleep Apnea	1028	94 (9.1)	13 (4.2)	81 (11.3)	0.0002
Comorbidities (*n*)	1028	3.5 ± 1.9	3.2 ± 1.8	3.7 ± 1.9	0.0002
Charlson index (points)	527	4.4 ± 2.8	3.98 ± 2.4	4.6 ± 3.0	0.009
Medications					
ACEIs or ARBs	1028	922 (89.7)	274 (88.7)	648 (90.1)	0.503
Beta-blockers	1028	787 (76.6)	224 (72.5)	563 (78.3)	0.045
MRAs	1028	684 (66.5)	202 (65.4)	482 (67.0)	0.614
Ivabradine	1028	91 (8.9)	21 (6.8)	70 (9.7)	0.151
Digoxin	1028	221 (21.5)	82 (26.5)	139 (19.3)	0.013
Diuretics	1028	917 (89.2)	281 (90.9)	636 (88.5)	0.273
Statins	1028	779 (75.8)	214 (69.3)	565 (78.6)	0.002
Antiplatelets	1028	618 (60.1)	154 (49.8)	464 (64.5)	<0.0001
Anticoagulants	1028	410 (39.9)	143 (46.3)	267 (37.1)	0.007
Laboratory parameters					
Hemoglobin (g/dL)	971	12.9 ± 1.7	12.4 ± 1.4	13.1 ± 1.7	<0.0001
eGFR (mL/min/1.73 m^2^)	573	60.0 (45.0–70.0)	60.0 (45.0–70.5)	60.0 (45.0–70.0)	0.542
Creatinine clearance <60 mL/min	573	259 (45.2)	85 (48.9)	174 (43.6)	0.274
NT-proBNP (pg/mL)	238	1345 (504–2390)	1348 (422–2333)	1333 (537–2411)	0.600
BNP (pg/mL)	154	216 (124–430)	211 (125–397)	232 (124–453)	0.258

Categorical variables are expressed as *n* and (%). Continuous variables are expressed using mean and standard deviation. Those continuous variables for which a normal distribution could not be assumed were expressed using median (Q1–Q3). ACEI: Angiotensin converting enzyme inhibitors, AMI: acute myocardial infarction, ARB: Angiotensin receptor blockers, BMI: body mass index, BNP: Brain natriuretic peptide, COPD: chronic obstructive pulmonary disease, eGFR: Estimated glomerular filtration rate, HF: heart failure, LVEF: left ventricular ejection fraction, MRA mineralocorticoid receptor antagonist, NTproBNP: N-terminal fraction of natriuretic propeptide type B, NYHA: New York Heart Association functional class, TIA: transient ischemic attack.

**Table 2:** Distribution of summary scores, dimensions, and domains of the Kansas City Cardiomyopathy Questionnaire (KCCQ) and EQ-5D quality of life questionnaires in the overall population studied and according to gender.

	*n*	Overall *n* = 1028	Women *n* = 309	Men *n* = 719	*p* Value
**KCCQ Subdomain Score**					
Physical Limitation	1023	61.1 ± 28.0	53.3 ± 29.1	64.5 ± 26.9	<0.0001
Symptom Stability	1022	59.5 ± 23.2	59.2 ± 24.0	59.7 ± 22.9	0.761
Symptom Frequency	1027	66.4 ± 26.0	60.8 ± 26.7	68.7 ± 25.4	<0.0001
Symptom Burden	1027	67.2 ± 26.0	61.6 ± 26.5	69.6 ± 25.5	<0.0001
Self-Efficacy	1026	69.1 ± 22.5	67.9 ± 22.8	69.6 ± 22.3	0.262
Quality of Life	1026	54.4 ± 24.1	50.8 ± 24.0	55.9 ± 24.0	0.002
Social Limitation	1018	61.6 ± 29.4	55.8 ± 29.5	64.1 ± 29.0	<0.0001
**KCCQ, Summary Scores**					
Overall Summary Score	1014	60.9 ± 24.5	55.0 ± 24.6	63.4 ± 24.0	<0.0001
Clinical Summary Score	1023	63.9 ± 25.1	57.3 ± 25.7	66.8 ± 24.4	<0.0001
Total Symptom Score	1027	66.8 ± 25.4	61.2 ± 25.9	69.2 ± 25.5	<0.0001
**EQ 5D, % patients reporting issues**					
Mobility	1001	581 (58.0)	212 (69.3)	369 (53.1)	<0.0001
Self-Care	1001	581 (58.0)	212 (69.3)	369 (53.1)	<0.0001
Usual Activities	1000	615 (61.5)	217 (70.9)	398 (57.3)	<0.0001
Pain/Discomfort	999	506 (50.7)	192 (62.7)	314 (45.3)	<0.0001
Anxiety/Depression	999	490 (59.0)	166 (54.2)	324 (46.8)	0.033
**EQ 5D Summary Scores**					
EQ 5D Index	993	0.65 ± 0.26	0.58 ± 0.26	0.67 ± 0.25	<0.0001
Visual analogue scale	1013	60.8 ± 20.0	57.5 ± 20.8	62.2 ± 19.4	0.001

**Table 3:** General linear multivariate models exploring interaction between gender and other clinical determinants in key variables with prognostic value in HF with reduced ejection fraction patients, over KCCQ global summary score.

	Women	Men	*p* Value
LVEF >30%	56.0 ± 1.5	63.3 ± 1.0	<0.001
LVEF <30%	52.8 ± 2.1	61.3 ± 1.4	0.003
*p* value for interaction	0.669 *		
Age <75 years	56.1 ± 1.8	64.2 ± 1.0	<0.001
Age >75 years	53.0 ± 1.7	60.3 ± 1.3	0.001
*p* value for interaction	0.766 *		
NYHA I-II	66.2 ± 1.8	72.7 ± 1.1	0.003
NYHA III-IV	42.1 ± 1.8	51.2 ± 1.2	<0.001
*p* value for interaction	0.364 *		
Comorbidities number <5	54.9 ± 1.8	64.4 ± 1.3	<0.001
Comorbidities number >5	55.3 ± 2.0	61.0 ± 1.2	0.005
*p* value for interaction	0.193 *		
Ischemic etiology: yes	55.3 ± 1.5	62.9 ± 1.3	<0.001
Ischemic etiology: no	54.5 ± 2.1	62.3 ± 1.0	<0.001
*p* value for interaction	0.959 *		
Recent admission: yes	58.3 ± 1.8	66.2 ± 1.1	<0.001
Recent admission: no	51.4 ± 1.7	59.0 ± 1.2	<0.001
*p* value for interaction	0.902 *		

LVEF: Left ventricular ejection fraction, NYHA: New York Heart Association functional class. Recent admission was defined as admission for heart failure within the last 6 months. * *p*-value of interaction between gender and variable.

## Supplementary Materials

The following are available online at https://www.mdpi.com/2077-0383/9/9/2825/s1, Table S1. Quality of life scores in HF patients for each of the KCCQ items in the overall population according to gender. Table S2. Distribution of quality of life scores in HF patients for each of the KCCQ items in the overall population according to gender. Table S3. Univariate and multivariate linear regression models evaluating the influence of gender and other clinical and biological determinants on disease-specific (KCCQ OSS) and generic (EQ-5D index and VAS) measures of HRQoL. Table S4 Univariate analysis using binary logistic regression exploring the clinical determinants of reporting limitations in each dimension of EQ-5D, stratified by gender (for women Table S4A, and for men Table S4B). Table S5. Multivariate adjusted binary logistic regression analyses using backwards methods evaluating the association of gender with the probability of reporting limitations in the 5 dimensions captured in the EQ-5D. Table S6. Adjusted binary logistic regression analyses exploring interaction effects between gender and clinical determinants on reported limitations in the 5 dimensions of the EQ-5D.
