# Supplementary material for: Gender Differences in Health-Related Quality of Life in Patients with Systolic Heart Failure: Results of the VIDA Multicenter Study
Source: J Clin Med. 2020 Aug 31;9(9):2825. doi: 10.3390/jcm9092825 (PMC7563299; doi:10.3390/jcm9092825)
Supplement: Supplementary file 1 [file jcm-09-02825-s001.pdf]

Journal of
Clinical Medicine

Supplementary Materials

Table 1. Quality of life scores in HF patients for each of the KCCQ items in the overall population according to gender.

KCCQ Item	Women<br>N = 309	Men<br>N = 719	p value
**Physical Limitation**			
Dressing yourself	3.9 ± 1.3	4.2 ± 1.1	<0.001
Showering/Bathing	3.7 ± 1.4	4.1 ± 1.2	<0.001
Walking 1 block on level ground	3.3 ± 1.4	3.8 ± 1.3	<0.001
Doing yardwork, housework or carrying groceries	2.6 ± 1.4	3.1 ± 1.4	<0.001
Climbing a flight of stairs without stopping	2.8 ± 1.4	3.3 ± 1.4	<0.001
Hurrying or jogging	2.1 ± 1.2	2.5 ± 1.4	<0.001
**Symptom Stability**			
Compared with 2 weeks ago, have your symptoms of heart failure (shortness of breath, fatigue, or ankle swelling) changed?	3.4 ± 1.0	3.4 ± 0.9	0.761
**Symptom Frequency**			
Over the past 2 weeks, how many times did you have swelling in your feet, ankles or legs when you woke up in the morning?	0.62 ± 0.35	0.71 ± 0.33	<0.001
Over the past 2 weeks, on average, how many times has fatigue limited your ability to do what you want?	0.53 ± 0.31	0.61 ± 0.30	<0.001
Over the past 2 weeks, on average, how many times has shortness of breath limited your ability to do what you wanted?	0.53 ± 0.29	0.63 ± 0.29	<0.001
Over the past 2 weeks, on average, how many times have you been forced to sleep sitting up in a chair or with at least 3 pillows to prop you up because of shortness of breath?	0.74 ± 0.31	0.80 ± 0.28	0.006
**Symptom Burden**			
Over the past 2 weeks, how much has swelling in your feet, ankles or legs bothered you?	3.7 ± 1.2	4.0 ± 1.2	0.001
Over the past 2 weeks, how much has your fatigue bothered you?	3.3 ± 1.2	3.6 ± 1.2	<0.001
Over the past 2 weeks, how much has your shortness of breath bothered you?	3.3 ± 1.2	3.6 ± 1.2	<0.001
**Self-Efficacy**			
How sure are you that you know what to do, or whom to call, if your heart failure gets worse?	3.7 ± 1.0	3.8 ± 1.0	0.732
How well do you understand what things you are able to do to keep your heart failure symptoms from getting worse? (for example, weighing yourself, eating a low salt diet etc.)	3.7 ± 1.0	3.8 ± 1.0	0.176
**Quality of Life**			
Over the past 2 weeks, how much has your heart failure limited your enjoyment of life?	3.2 ± 1.1	3.4 ± 1.1	0.006
If you had to spend the rest of your life with your heart failure the way it is right now, how would you feel about this?	2.8 ± 1.0	2.9 ± 1.1	0.150
Over the past 2 weeks, how often have you felt discouraged or down in the dumps because of your heart failure?	3.1 ± 1.1	3.4 ± 1.1	<0.001
**Social Limitation**			
Hobbies, recreational activities	3.4 ± 1.4	3.7 ± 1.4	0.002
Working or doing household chores	3.0 ± 1.6	3.3 ± 1.6	0.006
Visiting family or friends out of your home	3.2 ± 1.4	3.6 ± 1.4	<0.001
Intimate relationships with loved ones	2.8 ± 1.4	3.1 ± 1.5	0.004

Results expressed as score mean ± 1 standard error

Table 2. Frequency and distribution of quality of life scores in HF patients for each of the KCCQ items in the overall population according to gender.

KCCQ Item	Women *N* = 309	Men *N* = 719	*p* value
**Physical Limitation**			
Dressing yourself			<0.001
1-Extremely limited	11 (3.6%)	16 (2.2%)	
2-Quite a bit limited	43 (13.9%)	39 (5.4%)	
3-Moderately limited	47 (15.2%)	96 (13.4%)	
4-Slightly limited	70 (22.7%)	151 (21.0%)	
5-Not at all limited	135 (43.7%)	410 (57.0%)	
6- Limited for other reasons or did not do	3 (1.0%)	7 (1.0%)	
Showering/Bathing			<0.001
1-Extremely limited	21 (6.8%)	28 (3.9%)	
2-Quite a bit limited	48 (15.5%)	57 (7.9%)	
3-Moderately limited	51 (16.5%)	92 (12.8%)	
4-Slightly limited	66 (21.4%)	142 (19.7%)	
5-Not at all limited	119 (38.5%)	396 (55.1%)	
6- Limited for other reasons or did not do	4 (1.3%)	4 (0.6%)	
Walking 1 block on level ground			<0.001
1-Extremely limited	33 (10.7%)	33 (4.6%)	
2-Quite a bit limited	51 (16.5%)	96 (13.4%)	
3-Moderately limited	69 (22.3%)	111 (15.4%)	
4-Slightly limited	68 (22.0%)	193 (26.8%)	
5-Not at all limited	84 (27.2%)	175 (38.2%)	
6- Limited for other reasons or did not do	4 (1.3%)	11 (1.5%)	
Doing yardwork, housework or carrying groceries			<0.001
1-Extremely limited	73 (23.6%)	85 (11.8%)	
2-Quite a bit limited	62 (20.1%)	126 (17.5%)	
3-Moderately limited	63 (20.4%)	138 (19.2%)	
4-Slightly limited	66 (21.4%)	196 (27.3%)	
5-Not at all limited	31 (10.0%)	143 (19.9%)	
6- Limited for other reasons or did not do	14 (4.5%)	31 (4.3%)	
Climbing a flight of stairs without stopping			<0.001
1-Extremely limited	71 (23.0%)	81 (11.3%)	
2-Quite a bit limited	61 (19.7%)	126 (17.5%)	
3-Moderately limited	57 (18.4%)	133 (18.5%)	
4-Slightly limited	72 (23.3%)	202 (28.1%)	
5-Not at all limited	45 (14.6%)	164 (22.8%)	
6- Limited for other reasons or did not do	3 (1.0%)	13 (1.8%)	
Hurrying or jogging			<0.001
1-Extremely limited	131 (42.4%)	199 (27.7%)	
2-Quite a bit limited	70 (22.7%)	160 (22.3%)	
3-Moderately limited	50 (16.2%)	137 (19.1%)	
4-Slightly limited	40 (12.9%)	141 (19.6%)	
5-Not at all limited	13 (4.2%)	60 (8.3%)	
6- Limited for other reasons or did not do	5 (1.6%)	22 (3.1%)	
**Symptom Stability**			
Compared with 2 weeks ago, have your symptoms of heart failure (shortness of breath, fatigue, or ankle swelling) changed?			0.407
1-Much worse	8 (2.6%)	19 (2.7%)	
2-Slightly worse	36 (11.7%)	58 (8.1%)	
3-Not changed	143 (46.6%)	365 (51.0%)	
4-Slightly better	75 (24.4%)	173 (24.2%)	
5-Much better	45 (14.7%)	100 (14.0%)	
6-I've had no symptoms over the last 2 weeks	2 (0.6%)	4 (0.6%)	
**Symptom Frequency**			
Over the past 2 weeks, how many times did you have swelling in your feet, ankles or legs when you woke up in the morning?			0.007

1-Every morning	39 (12.6%)	62 (8.6%)	
2-Three or more times a week, but not every day	41 (13.3%)	69 (9.6%)	
3-One or two times a week	53 (17.2%)	104 (14.5%)	
4-Less than once a week	78 (25.2%)	175 (24.3%)	
5-Never over the past two weeks	98 (31.7%)	309 (43.0%)	
Over the past 2 weeks, on average, how many times has fatigue limited your ability to do what you want?			0,008
1-All of the time	28 (9.1%)	54 (7.5%)	
2-Several times per day	46 (14.9%)	67 (9.3%)	
3-At least once a day	45 (14.6%)	74 (10.3%)	
4-Three or more times per week but not every day	40 (12.9%)	82 (11.4%)	
5-One or two times per week	65 (21.0%)	179 (24.9%)	
6-Less than once a week	49 (15.9%)	143 (19.9%)	
7-Never over the past two weeks	36 (11.7%)	120 (16.7%)	
Over the past 2 weeks, on average, how many times has shortness of breath limited your ability to do what you wanted?			<0.001
1-All of the time	17 (5.5%)	29 (4.0%)	
2-Several times per day	52 (16.8%)	73 (10.2%)	
3-At least once a day	48 (15.5%)	69 (9.6%)	
4-Three or more times per week but not every day	41 (13.3%)	98 (13.6%)	
5-One or two times per week	77 (24.9%)	168 (23.4%)	
6-Less than once a week	41 (13.3%)	141 (19.6%)	
7-Never over the past two weeks	33 (10.7%)	141 (19.6%)	
Over the past 2 weeks, on average, how many times have you been forced to sleep sitting up in a chair or with at least 3 pillows to prop you up because of shortness of breath?			0.032
1-Every night	16 (5.2%)	30 (4.2%)	
2-Three or more times a week, but not every day	29 (9.4%)	42 (5.8%)	
3-One or two times a week	54 (17.5%)	91 (12.7%)	
4-Less than once a week	61 (19.7%)	160 (22.3%)	
5-Never over the past two weeks	149 (48.2%)	396 (55.1%)	
**Symptom Burden**			
Over the past 2 weeks, how much has swelling in your feet, ankles or legs bothered you?			0.012
1-Extremely bothersome	12 (3.9%)	15 (2.1%)	
2-Quite a bit bothersome	47 (15.2%)	81 (11.3%)	
3-Moderately bothersome	62 (20.1%)	123 (17.1%)	
4-Slightly bothersome	67 (21.7%)	130 (18.1%)	
5-Not at all bothersome	120 (38.8%)	367 (51.0%)	
6-I’ve had no swelling	1 (0.3%)	3 (0.4%)	
Over the past 2 weeks, how much has your fatigue bothered you?			0.002
1-Extremely bothersome	21 (6.8%)	24 (3.3%)	
2-Quite a bit bothersome	61 (19.7%)	108 (15.0%)	
3-Moderately bothersome	74 (23.9%)	150 (20.9%)	
4-Slightly bothersome	102 (33.0%)	247 (34.4%)	
5-Not at all bothersome	48 (15.5%)	182 (25.3%)	
6-I’ve had no fatigue	3 (1.0%)	8 (1.1%)	
Over the past 2 weeks, how much has your shortness of breath bothered you?			<0.001
1-Extremely bothersome	22 (7.1%)	22 (3.1%)	
2-Quite a bit bothersome	62 (20.1%)	119 (16.6%)	
3-Moderately bothersome	70 (22.7%)	131 (18.2%)	
4-Slightly bothersome	103 (33.3%)	238 (33.1%)	
5-Not at all bothersome	51 (16.5%)	203 (28.2%)	
6-I’ve had no shortness of breath	1 (0.3%)	6 (0.8%)	
**Self-Efficacy**			
How sure are you that you know what to do, or			0.561

whom to call, if your heart failure gets worse?			
1-Not at all sure	6 (1.9%)	12 (1.7%)	
2-Not very sure	33 (10.7%)	75 (10.4%)	
3-Somewhat sure	77 (24.9%)	153 (21.3%)	
4-Mostly sure	112 (36.2%)	294 (40.9%)	
5-Completely sure	81 (26.2%)	182 (25.3%)	
How well do you understand what things you are able to do to keep your heart failure symptoms from getting worse? (for example, weighing yourself, eating a low salt diet etc.)			0.178
1-Do not understand at all	4 (1.3%)	12 (1.7%)	
2-Do not understand very well	28 (9.1%)	54 (7.5%)	
3-Somewhat understand	95 (30.7%)	173 (24.1%)	
4-Mostly understand	115 (37.2%)	313 (43.5%)	
5-Completely understand	67 (21.7%)	165 (22.9%)	
**Quality of Life**			
Over the past 2 weeks, how much has your heart failure limited your enjoyment of life?			0.018
1-It has extremely limited my enjoyment of life	19 (6.1%)	38 (5.3%)	
2-It has limited my enjoyment of life quite a bit	83 (26.9%)	131 (18.2%)	
3-It has moderately limited my enjoyment of life	65 (21.0%)	171 (23.8%)	
4-It has slightly limited my enjoyment of life	105 (34.0%)	252 (35.0%)	
5-It has not limited my enjoyment of life at all	37 (12.0%)	125 (17.4%)	
If you had to spend the rest of your life with your heart failure the way it is right now, how would you feel about this?			0.183
1-Not at all satisfied	31 (10.0%)	77 (10.7%)	
2-Mostly dissatisfied	97 (31.4%)	183 (25.5%)	
3-Somewhat satisfied	96 (31.1%)	210 (29.2%)	
4-Mostly satisfied	72 (23.3%)	212 (29.5%)	
5-Completely satisfied	13 (4.2%)	34 (4.7%)	
Over the past 2 weeks, how often have you felt discouraged or down in the dumps because of your heart failure?			0.012
1-I felt that way all of the time	24 (7.8%)	32 (4.5%)	
2-I felt that way most of the time	64 (20.7%)	112 (15.6%)	
3-I occasionally felt that way	112 (36.2%)	253 (35.2%)	
4-I rarely felt that way	73 (23.6%)	191 (26.6%)	
5-I never felt that way	36 (11.7%)	129 (17.9%)	
**Social Limitation**			
Hobbies, recreational activities			0.005
1-Severely limited	21 (6.8%)	33 (4.6%)	
2-Limited quite a bit	54 (17.5%)	90 (12.5%)	
3-Moderately limited	53 (17.2%)	121 (16.8%)	
4-Slightly limited	92 (29.8%)	182 (25.3%)	
5-Did not limit at all	78 (25.2%)	268 (37.3%)	
6-Does not apply or did not do for other reasons	11 (3.6%)	25 (3.1%)	
Working or doing household chores			0.005
1-Severely limited	32 (10.4%)	66 (9.2%)	
2-Limited quite a bit	46 (14.9%)	100 (13.9%)	
3-Moderately limited	50 (16.2%)	127 (17.7%)	
4-Slightly limited	82 (26.5%)	146 (20.3%)	
5-Did not limit at all	64 (20.7%)	226 (31.4%)	
6-Does not apply or did not do for other reasons	35 (11.3%)	54 (7.5%)	
Visiting family or friends out of your home			<0.001
1-Severely limited	38 (12.3%)	53 (7.4%)	
2-Limited quite a bit	69 (22.3%)	94 (13.1%)	
3-Moderately limited	54 (17.5%)	140 (19.5%)	
4-Slightly limited	69 (22.3%)	160 (22.3%)	
5-Did not limit at all	74 (23.9%)	260 (36.2%)	
6-Does not apply or did not do for other reasons	5 (1.6%)	12 (1.7%)	
Intimate relationships with loved ones			<0.001
1-Severely limited	67 (21.7%)	111 (15.4%)	

2-Limited quite a bit	70 (22.7%)	121 (16.8%)
3-Moderately limited	56 (18.1%)	152 (21.1%)
4-Slightly limited	72 (23.3%)	151 (21.0%)
5-Did not limit at all	38 (12.3%)	156 (21.7%)
6-Does not apply or did not do for other reasons	6 (1.9%)	28 (3.9%)

Table 3. Univariate and multivariate linear regression models evaluating the influence of gender and other clinical and biological determinants on disease-specific (KCCQ OSS) and generic (EQ-5D index and VAS) measures of HRQoL.

Univariate Model

	**KCCQ OSS**			**EQ 5D Index**			**EQ 5D VAS**		
	**β coef.**	**Adj. R^2**	***p* value**	**β coef.**	**Adj. R^2**	***p* value**	**β coef.**	**Adj. R^2**	***p* value**
Gender, men/women	**-0.157**	**0.025**	**<0.0001**	**-0.169**	**0.029**	**<0.0001**	**-0.108**	**0.012**	**0.001**
Age, 1 year	-0.273	0.075	<0.0001	-0.285	0.081	<0.0001	-0.221	0.049	<0.0001
BMI, 1 Kg/m^2	-0.056	0.003	0.072	-0.084	0.007	0.008	-0.017	0.000	0.587
Systolic BP, 1 mmHg	-0.009	0.000	0.7693	0.001	0.000	0.977	0.042	0.002	0.182
Heart rate, 1 bpm	-0.105	0.011	0.001	-0.096	0.009	0.002	-0.060	0.004	0.055
NYHA functional class, I-II/III-IV	-0.561	0.315	<0.0001	-0.464	0.215	<0.0001	-0.454	0.206	<0.0001
LVEF, 1 % unity	0.152	0.023	<0.0001	0.128	0.016	<0.0001	0.163	0.027	<0.0001
Comorbidities number, 1 point	-0.307	0.094	<0.0001	-0.316	0.100	<0.0001	-0.221	0.049	<0.0001
Ischemic etiology, no/yes	-0.070	0.005	0.026	-0.086	0.007	0.007	-0.051	0.003	0.104
Kidney failure, no/yes	-0.242	0.059	<0.0001	-0.209	0.044	<0.0001	-0.202	0.041	<0.0001
Hypertension	-0.097	0.009	0.002	-0.125	0.016	<0.0001	-0.039	0.002	0.211
Atrial Fibrillation, no/yes	-0.150	0.022	<0.0001	-0.173	0.030	<0.0001	-0.136	0.019	<0.0001
Diabetes mellitus, no/yes	-0.149	0.022	<0.0001	-0.165	0.027	<0.0001	-0.104	0.011	0.001
Anemia, no/yes	-0.178	0.032	<0.0001	-0.204	0.041	<0.0001	-0.143	0.021	<0.0001
Admission Service, CAR/IM	-0.184	0.034	<0.0001	-0.197	0.039	<0.0001	-0.185	0.034	<0.0001
Recent admission, no/yes	-0.256	0.066	<0.001	-0.199	0.040	<0.0001	-0.191	0.036	<0.0001
Time from diagnosis, years	-0.074	0.005	0.025	-0.060	0.004	0.070	-0.074	0.005	0.025
Optimal treatment, yes/no	0.040	0.002	0.208	0.061	0.004	0.055	0.046	0.002	0.142

BMI: Body Mass Index, BP: Blood Pressure, NYHA: New York Heart Association Functional Class, LVEF: Left Ventricular Ejection Fraction, CAR: Cardiology, IM: Internal Medicine.

Multivariate model (backwards stepwise methods)

	**KCCQ OSS**		**EQ 5D Index**		**EQ 5D VAS**	
	**β coef.**	***p* value**	**β coef.**	***p* value**	**β coef.**	***p* value**

Gender, men/women	**-0.144**	**<0.0001**	**-0.157**	**<0.0001**	**-0.106**	**0.0005**
Age, 1 year	-0.113	<0.0001	-0.135	<0.0001	-0.094	0.003
BMI, 1 Kg/m^2						
Systolic BP, 1 mmHg						
Heart rate, 1 bpm						
NYHA functional class, I-II/III-IV	-0.445	<0.0001	-0.328	<0.0001	-0.340	<0.0001
LVEF, 1 % unity					0.066	0.035
Comorbidities number, 1 point	-0.139	<0.0001	-0.186	<0.0001	-0.084	0.009
Ischemic etiology, no/yes						
Kidney failure, no/yes						
Hypertension						
Atrial Fibrillation, no/yes						
Diabetes mellitus, no/yes						
Anemia, no/yes						
Admission Service, CAR/IM	-0.100	0.0003	-0.109	<0.0001	-0.120	<0.0001
Recent admission, no/yes	-0.149	<0.0001	-0.099	0.001	-0.100	0.001
Time from diagnosis, years						
Optimal treatment, yes/no						
Adjusted R^2 for each model	0.405		0.322		0.255	

BMI: Body Mass Index, BP: Blood Pressure, NYHA: New York Heart Association Functional Class, LVEF: Left Ventricular Ejection Fraction, CAR: Cardiology, IM: Internal Medicine.

Table 4. Univariate analysis using binary logistic regression exploring the clinical determinants of reporting limitations in each dimension of EQ-5D, stratified by gender (for women Supplementary Table 4A, and for men Supplementary Table 4B).

Table 4A (Women)

	**Mobility**			**Self-Care**			**Usual Activities**		
	**OR**	**95% IC**	***p* value**	**OR**	**95% IC**	***p* value**	**OR**	**95% IC**	***p* value**
Age, ≥75 years vs. <75 years	2.6	1.6-4.4	0.0002	2.6	1-6-4.4	<0.001	1.9	1.1-3.1	0.014
BMI ≥ 30 Kg/m^2	1.6	0.9-2.7	0.106	1.6	0.9-2.7	0.106	1.9	1.1-3.4	0.023
Systolic BP ≤110 mmHg	0.8	0.5-1.4	0.474	0.8	0.5-1.4	0.474	0.6	0.3-1.0	0.042
Heart rate ≥80 bpm vs <80	1.6	1.0-2.8	0.067	1.6	1.0-2.8	0.067	1.2	0.7-2.1	0.438
NYHA functional class III-IV Vs I-II	4.9	2.8-8.6	<0.0001	4.9	2.8-8.6	<0.001	5.2	2.9-9.4	<0.001

LVEF ≤30 % vs >30%	2.1	1.2-3.7	0.013	2.1	1.2-3.7	0.013	1.3	0.7-2.2	0.404
Comorbidities number ≥5 vs <5	1.7	1.0-2.8	0.031	1.7	1.1-2.9	0.031	2.1	1.3-3.6	0.004
Ischemic etiology yes vs no	2.1	1.2-3.6	0.011	2.1	1.2-3.6	0.011	1.5	0.8-2.5	0.174
Chronic kidney failure, yes vs no	1.0	1.0-3.7	0.054	1.9	1.0-3.7	0.054	3.4	1.6-7.6	0.002
Hypertension yes vs no	2.4	1.4-4.2	0.002	2.4	1.4-4.2	0.002	1.3	0.7-2.3	0.378
Atrial Fibrillation yes vs no	1.2	0.8-2.0	0.402	1.2	0.8-2.0	0.402	1.6	0.9-2.6	0.083
Diabetes mellitus yes vs no	1.9	1.2-3.2	0.011	1.9	1.2-3.1	0.011	1.7	1.0-2.8	0.044
Anemia yes vs no	1.1	0.6-2.0	0.657	1.1	0.6-2.0	0.657	1.2	0.7-2.1	0.516
Admission Service IM Vs CAR	1.5	0.9-2.4	0.123	1.5	0.9-2.5	0.123	1.1	0.6-1.8	0.787
Recent admission yes vs no	1.7	1.1-2.8	0.029	1.7	1.1-2.8	0.029	1.9	1.2-3.2	0.010
Time from diagnosis ≥1 year vs <1 year	1.5	0.9-1.7	0.143	1.5	0.9-2.7	0.143	1.2	0.7-2.2	0.522
Optimal treatment yes vs no	1.1	0.7-1.8	0.702	1.1	0.7-1.8	0.702	1.1	0.7-1.8	0.767

	Pain/ Discomfort			Anxiety/ Depression		
	**OR**	**95% IC**	***p* value**	**OR**	**95% IC**	***p* value**
Age, ≥75 years vs. <75 years	1.5	1.0-2.4	0.070	1.2	0.7-1.8	0.481
BMI ≥ 30 Kg/m²	2.2	1.3-3.7	0.004	1.7	1.1-2.8	0.031
Systolic BP ≤110 mmHg	0.6	0.3-1.0	0.035	1.2	0.7-2.1	0.465
Heart rate ≥80 bpm vs <80	1.5	0.9-2.4	0.138	1.2	0.7-1.9	0.457
NYHA functional class III-IV Vs I-II	3.6	2.1-6.0	<0.001	5.2	3.1-8.6	<0.001
LVEF ≤30 % vs >30%	1.1	0.6-1.8	0.817	2.0	1.2-3.2	0.009
Comorbidities number ≥5 vs <5	2.0	1.2-3.1	0.005	1.6	1.0-2.5	0.048
Ischemic etiology yes vs no	1.3	0.8-2.2	0.284	1.2	0.7-1.9	0.501
Chronic kidney failure, yes vs no	2.0	1.1-3.7	0.031	2.1	1.2-3.8	0.013
Hypertension yes vs no	1.8	1.0-3.0	0.040	1.4	0.8-2.4	0.225
Atrial Fibrillation yes vs no	1.6	1.0-2.6	0.052	1.2	0.7-1.8	0.479
Diabetes mellitus yes vs no	1.5	0.9-2.3	0.122	1.8	1.1-2.8	0.016
Anemia yes vs no	1.5	0.9-2.7	0.120	1.5	0.9-2.5	0.145

Admission Service IM Vs CAR	1.7	1.1-2.8	0.024	1.2	0.8-1.9	0.443
Recent admission yes vs no	1.4	0.9-2.3	0.141	1.7	1.1-2.6	0.026
Time from diagnosis ≥1 year vs <1 year	0.9	0.5-1.6	0.775	0.8	0.5-1.4	0.481
Optimal treatment yes vs no	1.0	0.6-1.6	0.996	1.0	0.6-1.6	0.938

OR: Odds Ratio. BMI: Body Mass Index, BP: Blood Pressure, NYHA: New York Heart Association Functional Class, LVEF: Left Ventricular Ejection Fraction, CAR: Cardiology, IM: Internal Medicine.

Table 4B (Men)

	**Mobility**			**Self-Care**			**Usual Activities**		
	**OR**	**95% IC**	***p* value**	**OR**	**95% IC**	***p* value**	**OR**	**95% IC**	***p* value**
Age, ≥75 years vs. <75 years	2.6	1.9-3.6	<0.001	2.6	1.9-3.6	<0.001	2.0	1.5-2.8	<0.001
BMI ≥ 30 Kg/m^2	1.4	0.9-2.0	0.101	1.4	0.9-2.0	0.101	1.0	0.7-1.4	0.956
Systolic BP ≤110 mmHg	1.0	0.7-1.4	0.961	1.0	0.7-1.4	0.961	1.0	0.7-1.4	0.918
Heart rate ≥80 bpm vs <80	1.3	0.9-1.8	0.133	1.3	0.9-1.8	0.133	1.7	1.2-2.4	0.002
NYHA functional class III-IV Vs I-II	4.8	3.4-6.6	<0.001	4.8	3.4-6.6	<0.001	6.4	4.5-9.1	<0.001
LVEF ≤30 % vs >30%	1.4	1.0-2.0	0.030	1.4	1.0-2.0	0.030	1.1	0.8-1.5	0.568
Comorbidities number ≥5 vs <5	2.7	2.0-3.6	<0.001	2.7	2.0-3.6	<0.001	2.5	1.8-3.4	<0.001
Ischemic etiology yes vs no	1.3	1.0-1.8	0.078	1.3	1.0-1.8	0.078	1.5	1.1-2.0	0.009
Chronic kidney failure, yes vs no	3.3	2.2-4.8	<0.001	3.3	2.2-4.8	<0.001	2.2	1.5-3.2	<0.001
Hypertension yes vs no	1.9	1.3-2.8	0.001	1.9	1.3-2.8	0.001	1.4	0.9-2.0	0.105
Atrial Fibrillation yes vs no	2.0	1.5-2.8	<0.001	2.0	1.5-2.8	<0.001	1.7	1.2-2.3	0.001
Diabetes mellitus yes vs no	2.0	1.4-2.7	<0.001	2.0	1.4-2.7	<0.001	1.8	1.3-2.5	<0.001
Anemia yes vs no	2.8	1.8-4.3	<0.001	2.8	1.8-4.2	<0.001	2.3	1.5-3.7	<0.001
Admission Service IM Vs CAR	1.8	1.3-2.6	<0.001	1.8	1.3-2.6	<0.001	1.4	1.0-2.0	0.025
Recent admission yes vs no	2.1	1.5-2.8	<0.001	2.1	1.5-2.8	<0.001	2.2	1.6-3.0	<0.001
Time from diagnosis ≥1 year vs <1 year	1.4	1.0-2.1	0.044	1.4	1.0-2.1	0.044	1.4	1.0-2.0	0.079
Optimal treatment yes vs no	0.7	0.5-1.0	0.039	0.7	0.5-1.0	0.039	0.9	0.7-1.2	0.453

	**Pain/ Discomfort**			**Anxiety/ Depression**		

	OR	95% IC	*p* value	OR	95% IC	*p* value
Age, ≥75 years vs. <75 years	1.8	1.3-2.5	<0.001	1.0	0.7-1.3	0.979
BMI ≥ 30 Kg/m^2	1.6	1.1-2.4	0.010	1.1	0.7-1.5	0.769
Systolic BP ≤110 mmHg	0.8	0.6-1.1	0.231	1.6	1.2-2.3	0.005
Heart rate ≥80 bpm vs <80	1.4	1.0-2.0	0.028	1.5	1.1-2.0	0.024
NYHA functional class III-IV Vs I-II	3.2	2.3-4.4	<0.001	3.6	2.6-4.9	<0.001
LVEF ≤30 % vs >30%	1.0	0.8-1.4	0.827	1.9	1.4-2.6	<0.001
Comorbidities number ≥5 vs <5	2.4	1.7-3.2	<0.001	2.1	1.6-2.9	<0.001
Ischemic etiology yes vs no	1.2	0.9-1.6	0.358	1.8	1.3-2.4	<0.001
Chronic kidney failure, yes vs no	1.9	1.3-2.7	<0.001	2.1	1.5-3.0	<0.001
Hypertension yes vs no	1.9	1.3-2.8	0.001	1.2	0.8-1.7	0.318
Atrial Fibrillation yes vs no	2.0	1.5-2.7	<0.001	1.6	1.2-2.2	0.002
Diabetes mellitus yes vs no	1.6	1.2-2.2	0.002	1.8	1.3-2.4	<0.001
Anemia yes vs no	1.9	1.3-2.9	0.002	2.0	1.3-3.1	0.001
Admission Service IM Vs CAR	1.8	1.3-2.4	<0.001	1.4	1.0-1.9	0.053
Recent admission yes vs no	1.8	1.3-2.4	<0.001	1.8	1.3-2.5	<0.001
Time from diagnosis ≥1 year vs <1 year	1.1	0.8-1.6	0.553	0.9	0.6-1.2	0.462
Optimal treatment yes vs no	0.8	0.6-1.0	0.062	0.8	0.6-1.0	0.114

OR: Odds Ratio. BMI: Body Mass Index, BP: Blood Pressure, NYHA: New York Heart Association Functional Class, LVEF: Left Ventricular Ejection Fraction, CAR: Cardiology, IM: Internal Medicine.

Table 5. Multivariate adjusted binary logistic regression analyses using backwards methods evaluating the association of gender with the probability of reporting limitations in the 5 dimensions captured in the EQ-5D (mobility, usual activities, self-care, pain/discomfort or anxiety/depression). We present odds ratios and 95% confidence intervals along with p-values comparing women with men (reference category) of reporting any limitation in each of the dimensions of the EQ-5D questionnaire. The models were adjusted for clinical covariates that showed significant association with HRQoL in univariate analyses. .

	**Mobility**			**Self-Care**			**Usual Activities**		
	**OR**	**95% IC**	***p* value**	**OR**	**95% IC**	***p* value**	**OR**	**95% IC**	***p* value**
Gender, men vs women	**2.3**	**1.6-3.2**	**<0.0001**	**2.3**	**1.6-3.2**	**<0.0001**	**1.8**	**1.3-2.6**	**0.001**
Age, ≥75 years vs. <75 years	2.0	1.4-2.7	<0.0001	2.0	1.4-2.7	<0.0001	1.6	1.2-2.2	0.004
Systolic BP ≤110 mmHg	0.7	0.5-1.0	0.045	0.7	0.5-1.0	0.045	0.6	0.4-0.8	0.002

Heart rate ≥80 bpm vs <80	1.0	0.7-1.5	0.843	1.0	0.7-1.5	0.843	1.2	0.8-1.7	0.427
NYHA functional class III-IV Vs I-II	3.4	2.5-4.8	<0.0001	3.4	2.5-4.8	<0.0001	5.0	3.6-7.1	<0.0001
LVEF ≤30 % vs >30%	1.3	0.9-1.9	0.110	1.3	0.9-1.9	0.110	0.8	0.5-1.1	0.133
Comorbidities number ≥5 vs <5	0.9	0.6-1.4	0.634	0.9	0.6-1.4	0.634	1.2	0.8-1.8	0.269
Ischemic etiology yes vs no	1.2	0.9-1.7	0.279	1.2	0.9-1.7	0.279	1.1	0.8-1.6	0.433
Chronic kidney failure, yes vs no	1.6	1.1-2.4	0.023	1.6	1.1-2.4	0.023	1.3	0.9-2.0	0.158
Hypertension yes vs no	1.6	1.1-2.3	0.019	1.6	1.1-2.3	0.019	1.0	0.7-1.5	0.991
Atrial Fibrillation yes vs no	1.2	0.9-1.7	0.260	1.2	0.9-1.7	0.260	1.0	0.7-1.4	0.844
Diabetes mellitus yes vs no	1.4	1.0-2.0	0.031	1.4	1.0-2.0	0.031	1.5	1.1-2.1	0.009
Anemia yes vs no	1.0	0.6-1.6	0.878	1.0	0.6-1.6	0.878	1.1	0.7-1.8	0.700
Admission Service IM Vs CAR	1.3	0.9-1.8	0.114	1.3	0.9-1.8	0.114	1.0	0.7-1.4	0.888
Recent admission yes vs no	1.6	1.2-2.3	0.003	1.6	1.2-2.3	0.003	1.6	1.1-2.2	0.006
Time from diagnosis ≥1 year vs <1 year	1.5	1.1-2.2	0.023	1.5	1.1-2.2	0.023	1.4	1.0-2.0	0.070
Optimal treatment yes vs no	0.8	0.6-1.2	0.324	0.8	0.6-1.2	0.324	1.1	0.8-1.5	0.697

	**Pain/ Discomfort**			**Anxiety/ Depression**		
	**OR**	**95% IC**	***p* value**	**OR**	**95% IC**	***p* value**
Gender, men vs women	**2.0**	**1.4-2.8**	<0.0001	**1.5**	**1.1-2.0**	**0.021**
Age, ≥75 years vs. <75 years	1.5	1.1-2.0	0.017	0.8	0.6-1.1	0.130
Systolic BP ≤110 mmHg	0.6	0.4-0.9	0.009	1.4	1.0-2.0	0.042
Heart rate ≥80 bpm vs <80	1.2	0.8-1.6	0.311	1.0	0.7-1.4	0.952
NYHA functional class III-IV Vs I-II	2.8	2.2-3.8	<0.0001	3.0	2.2-4.1	<0.0001
LVEF ≤30 % vs >30%	0.9	0.6-1.2	0.491	1.6	1.1-2.1	0.006
Comorbidities number ≥5 vs <5	1.4	1.0-2.0	0.044	1.0	0.7-1.5	0.895
Ischemic etiology yes vs no	0.9	0.6-1.3	0.582	1.3	0.9-1.7	0.143
Chronic kidney failure, yes vs no	1.1	0.7-1.6	0.682	1.4	0.9-2.0	0.098
Hypertension yes vs no	1.4	1.0-2.1	0.079	1.2	0.8-1.7	0.336
Atrial Fibrillation yes	1.6	1.2-2.2	0.002	1.2	0.9-1.7	0.190

vs no						
Diabetes mellitus yes vs no	1.1	0.8-1.5	0.626	1.6	1.2-2.2	0.002
Anemia yes vs no	1.0	0.7-1.6	0.830	1.1	0.7-1.7	0.539
Admission Service IM Vs CAR	1.3	0.9-1.8	0.108	1.0	0.7-1.3	0.894
Recent admission yes vs no	1.3	1.0-1.8	0.079	1.2	0.9-1.6	0.282
Time from diagnosis ≥1 year vs <1 year	1.0	0.7-1.4	0.963	0.7	0.5-1.0	0.075
Optimal treatment yes vs no	1.0	0.7-1.4	0.981	0.7	0.5-0.9	0.020

OR: Odds Ratio. BMI: Body Mass Index, BP: Blood Pressure, NYHA: New York Heart Association Functional Class, LVEF: Left Ventricular Ejection Fraction, CAR: Cardiology, IM: Internal Medicine.

Table 6. Adjusted binary logistic regression analyses exploring interaction effects between gender and clinical determinants on reported limitations in the 5 dimensions of the EQ-5D (mobility, usual activities, self-care, pain/discomfort or anxiety/depression). We present p-values for the interaction term gender by each clinical variable. The models were adjusted for clinical covariates that showed significant association with HRQoL in univariate analyses.

	**Mobility**	**Self-Care**	**Usual Activities**	**Pain/ Discomfort**	**Anxiety/ Depression**
**Interaction Gender * Variable**	***P-value* for interaction**	***P-value* for interaction**	***P-value* for interaction**	***P-value* for interaction**	***P-value* for interaction**
Age, ≥75 years vs <75 years	0.163	0.163	0.276	0.418	0.281
BMI ≥ 30 Kg/m^2	0.768	0.768	0.159	0.248	0.212
Systolic BP ≤110 mmHg	0.612	0.612	0.203	0.213	0.219
Heart rate ≥80 bpm vs <80	0.443	0.443	0.704	0.175	0.788
NYHA functional class III-IV Vs I-II	0.862	0.862	0.484	0.944	0.056
LVEF ≤30 % vs >30%	0.286	0.286	0.624	0.937	0.824
Comorbidities number ≥5 vs <5	0.055	0.055	0.201	0.454	0.149
Ischemic etiology yes vs no	0.124	0.124	0.911	0.979	0.330
Chronic kidney failure, yes vs no	0.412	0.412	0.306	0.930	0.923
Hypertension yes vs no	0.931	0.931	0.398	0.360	0.865
Atrial Fibrillation yes vs no	0.044	0.044	0.491	0.845	0.282
Diabetes mellitus yes vs no	0.554	0.554	0.406	0.966	0.620
Anemia yes vs no	0.014	0.014	0.098	0.928	0.457
Admission	0.945	0.945	0.995	0.697	0.728

Service IM Vs CAR					
Recent admission yes vs no	0.597	0.597	0.643	0.397	0.815
Time from diagnosis ≥1 year vs <1 year	0.716	0.716	0.843	0.893	0.808
Optimal treatment yes vs no	0.131	0.131	0.498	0.170	0.798

BMI: Body Mass Index, BP: Blood Pressure, NYHA: New York Heart Association Functional Class, LVEF: Left Ventricular Ejection Fraction, CAR: Cardiology, IM: Internal Medicine.
